# Cardio-Oncoimmunology: Cardiac Toxicity, Cardiovascular Hypersensitivity, and Kounis Syndrome

**DOI:** 10.3390/life14030400

**Published:** 2024-03-18

**Authors:** Nicholas G. Kounis, Ming-Yow Hung, Cesare de Gregorio, Virginia Mplani, Christos Gogos, Stelios F. Assimakopoulos, Panagiotis Plotas, Periklis Dousdampanis, Sophia N. Kouni, Anastasopoulou Maria, Grigorios Tsigkas, Ioanna Koniari

**Affiliations:** 1 Department of Medicine, Division of Cardiology, University Hospital of Patras, 26500 Patras, Greece; grigoriostsigkas@gmail.com; 2 Division of Cardiology, Department of Internal Medicine, Shuang Ho Hospital, Taipei Medical University, New Taipei City 23561, Taiwan; 3 Taipei Heart Institute, Taipei Medical University, Taipei City 110301, Taiwan; 4 Division of Cardiology, Department of Internal Medicine, School of Medicine, College of Medicine, Taipei Medical University, Taipei City 110301, Taiwan; 5 Department of Clinical and Experimental Medicine, University of Messina Medical School, 98122 Messina, Italy; cesare.degregorio@unime.it; 6 Intensive Care Unit, Patras University Hospital, 26504 Patras, Greece; 7 Department of Cardiology, Papageorgiou General Hospital, Nea Efkarpia, 56403 Thessaloniki, Greece; gogos-grivas@hotmail.com; 8 First University Cardiology Department, AHEPA General Hospital, Aristotle University of Thessaloniki, 54124 Thessaloniki, Greece; 9 Division of Infectious Diseases, Department of Internal Medicine, Medical School, University of Patras, University Hospital of Patras, 26504 Patras, Greece; 10 Department of Speech Therapy, University of Patras, 26504 Patras, Greece; 11 Department of Nephrology, Saint Andrews State General Hospital, 26221 Patras, Greece; 12 Speech Therapy Practice, Queen Olgas Square, 26221 Patras, Greece; 13 Department of Cardiology, General Hospital of Aigio, 25100 Aigio, Greece; anastmaria89@hotmail.com; 14 Cardiology Department, Lancashire Cardiac Center, Blackpool Teaching Hospitals, Blackpool FY3 8NP, UK; iokoniari@yahoo.gr; 15 Liverpool Center of Cardiovascular Science, Liverpool Heart and Chest Hospital, Liverpool L14 3PE, UK

**Keywords:** cardio-oncology, cardiovascular hypersensitivity, cardiac toxicity, coronary spasm, Kounis syndrome

## Abstract

Cancer therapy can result in acute cardiac events, such as coronary artery spasm, acute myocardial infarction, thromboembolism, myocarditis, bradycardia, tachyarrhythmias, atrio-ventricular blocks, QT prolongation, torsades de pointes, pericardial effusion, and hypotension, as well as chronic conditions, such as hypertension, and systolic and diastolic left ventricular dysfunction presenting clinically as heart failure or cardiomyopathy. In cardio-oncology, when referring to cardiac toxicity and cardiovascular hypersensitivity, there is a great deal of misunderstanding. When a dose-related cardiovascular side effect continues even after the causative medication is stopped, it is referred to as a cardiotoxicity. A fibrotic response is the ultimate outcome of cardiac toxicity, which is defined as a dose-related cardiovascular adverse impact that lasts even after the causative treatment is stopped. Cardiotoxicity can occur after a single or brief exposure. On the other hand, the term cardiac or cardiovascular hypersensitivity describes an inflammatory reaction that is not dose-dependent, can occur at any point during therapy, even at very low medication dosages, and can present as Kounis syndrome. It may also be accompanied by anti-drug antibodies and tryptase levels. In this comprehensive review, we present the current views on cardiac toxicity and cardiovascular hypersensitivity, together with the reviewed cardiac literature on the chemotherapeutic agents inducing hypersensitivity reactions. Cardiac hypersensitivity seems to be the pathophysiologic basis of coronary artery spasm, acute coronary syndromes such as Kounis syndrome, and myocarditis caused by cancer therapy.

## 1. Introduction

With the current prolonged life span of patients with cancer, the adverse effects of chemotherapy, especially cardiovascular disease, have gained enormous attention. Indeed, the incidence of cardiovascular events, such as cardiac injury, cardiovascular toxicity, or cardiovascular hypersensitivity, is higher than the recurrence rate of malignant tumors [1]. Whereas ischemic heart disease constitutes the most common cause of cardiac failure worldwide, the cardiovascular complications of chemotherapy have tended to become the second most common leading cause of heart failure. Therefore, several mechanisms, including ischemic heart disease, have been proposed to explain the association between anticancer treatments and myocardial disease [2]. Cardiovascular dysfunction or cardiac function worsening during chemotherapy might be attributed to chemotherapeutic drugs or to radiation therapy. Indeed, in a recent report concerning patients with left-sided breast cancer who received a higher mean heart dose of radiotherapy, the risk of ischemic heart disease was raised by 6.2% per Gy (gray Units of ionizing radiation dose, hazard ratio 1.062, 95% confidence interval 1.01–1.12; *p* = 0.012) [3]. Additional significant risk factors were age, chronic kidney disease, and hyper-lipidaemia. The authors proposed that the left ventricle receiving 25 Gy (LV V25) ≥ 4% is the best parameter in predicting major myocardial ischemic events and was superior to the mean heart dose of radiotherapy. Better heart protection in breast cancer radiotherapy could be achieved using the above dose of radiotherapy, though further validation studies are warranted. Cardiovascular deterioration can manifest as acute cardiac events, including coronary spasm, acute myocardial infarction, hypotension, cardiac arrhythmias (bradycardia, tachyarrhythmias, atrio-ventricular blocks, QT prolongation, torsades de pointes), pericarditis, myocarditis, pericardial effusion, and thrombo-embolism. Moreover, chronic conditions, such as hypertension, and systolic and diastolic left ventricular dysfunction presenting clinically as heart failure or cardiomyopathy, are additional pathologies. Drugs can affect the cardiovascular system either through direct effects on cardiac myocytes, resulting in cardiomyopathy, or through an indirect impact, such as hypertension, subsequently increasing the risk of heart disease [4]. The two main public health issues that have the biggest effects on society and the economy are cancer and cardiovascular disease. Research in the area of long-term cancer survivorship care is progressing. Many survivors will endure a number of long-term consequences from their cancer and treatment. After cancer treatment, a cardiovascular risk assessment at the end of the course will determine which patients need long-term cardiology follow-up beyond the first year. Patients with cancer who are asymptomatic but have a high risk of cardiovascular events in the future need to be monitored for a long time.

In this review, we provide an overview of cardiovascular dysfunction or worsening of cardiac function and cardiovascular hypersensitivity associated with the use of anticancer drugs.

## 2. Cardiac Toxicity

The term “cardiotoxicity,” which refers to all the side effects of cancer therapy, is widely used in cardiovascular society. When a dose-related cardiovascular side effect continues even after the causing treatment is stopped, it is referred to as cardiotoxicity. Acute toxicity is the term used to describe the negative effects from a single or brief exposure. Heart toxicity ultimately results in a fibrotic response that needs to be confirmed histologically—a process that has not been conducted much up to this point [5]. The phrase “cardiac dysfunction related to cancer treatment” has been used interchangeably. There is still much to learn about the definition, characterization, and pathophysiology of cardiac dysfunction during chemotherapy. The term cardiotoxicity lacks consensus in medical societies, especially when this term is used to describe the acute adverse effects of chemotherapeutic monoclonal antibodies. Broadly speaking, it encompasses both the quantity of the material to which the body is exposed and the mode of exposure, such as skin absorption, oral ingestion, or respiratory tract inhalation. Subchronic toxicity is the ability of a toxic substance to cause effects for more than one year but less than the lifetime of the exposed organism. Chronic toxicity refers to the ability of a substance or mixture of substances to exert harmful effects over a prolonged period.

The definition of cardiotoxicity is important and has practical implications regarding how patients are managed. Unfortunately, there is no universal definition of cardiotoxicity. Although the definitions reported in clinical trials differ, they all thematically define cardiotoxicity using a progressive decrease in left ventricular ejection fraction. Various organizations have defined cardiotoxicity differently using different threshold changes in left ventricular ejection fraction [6].

A more detailed definition has been proposed by the Evaluation Committee that oversees the clinical trials of trastuzumab, including one or more of the following: global or more severe diaphragmatic cardiomyopathy with reduced left ventricular ejection fraction; symptoms of heart failure; and cardiac signs, including an audible third heart sound associated with a galloping rhythm, tachycardia, or both.

**Figure 1:**
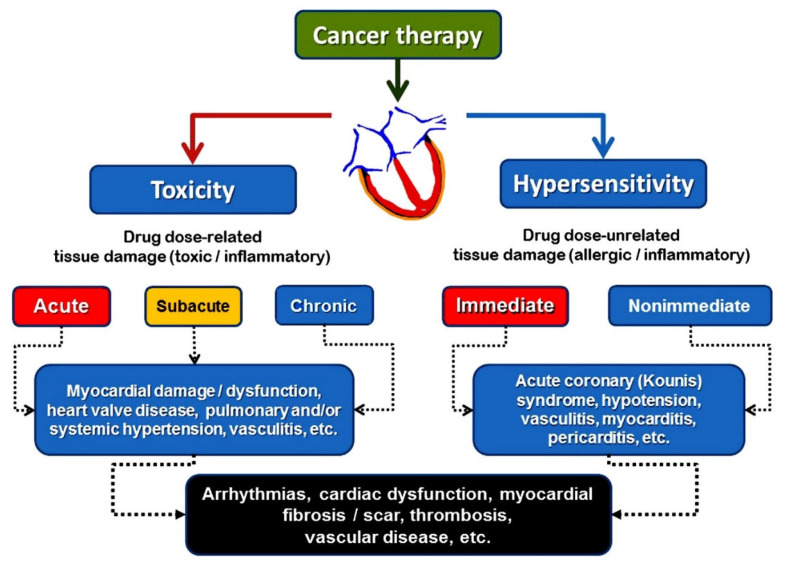
Different pathophysiological mechanisms leading to cardiac damage during cancer therapy. Further explanation in text.

Regarding echocardiography, the reduction in left ventricular ejection fraction is a mainstay index for deciding on drug-induced myocardial dysfunction. However, there is no agreement regarding the cut-off index, ranging from ≤5% to ≤55% from the baseline values [7]. The European Society of Cardiology’s current guidelines [8] for the non-invasive diagnosis of amyloid light-chain cardiac amyloidosis include specific strong recommendations on the use of a multiparametric echocardiographic score of ≥8 to define left ventricular functional impairment during cancer therapy, as follows:

1. relative wall thickness > 0.6 (3 points),
2. apex-to-basis ratio of systolic longitudinal strain >2.9 (3 points),
3. tricuspid annular plane systolic excursion, used as an objective measure of right-ventricular dysfunction (TAPSE) ≤ 19 mm (2 points),
4. left ventricular global longitudinal strain (GLS) ≥−13% (1 point) and
5. E/e’ ratio, which is a parameter for diastolic function assessment that is frequently used for heart failure with preserved ejection fraction evaluation (E/e’ ratio) > 11 (1 point) [8]. Cardiovascular hypersensitivity seems to be the pathophysiologic basis of acute coronary syndromes, such as coronary vasospasm, acute myocardial infarction, Kounis syndrome, and myocarditis, caused by cancer therapy (Figure 1).

## 3. Cardiovascular Hypersensitivity

An inflammatory response known as cardiovascular hypersensitivity is not dose-dependent. It can occur at any point during treatment, even at very low drug dosages, and may be accompanied by tryptase and anti-drug antibodies. IgE antibodies are involved in anti-drug antibodies in hypersensitivity reactions, but the IgG isotype may also be involved. It is true that certain chemotherapy medications have been linked to IgE-mediated hypersensitivity reactions. It is possible that the classical skin manifestations are absent, and the tryptase measurements are misleading. Doctors need to be informed that there is a formula that determines the minimum acute elevation in tryptase level that is clinically significant (peak mast cell tryptase should be >1.2× baseline tryptase + 2 mg/L) [9].

In severe hypersensitivity (anaphylaxis), the skin manifestations may be absent, which can render the diagnosis difficult [10]. Severe hypotension caused by decreased cardiac output from volume loss and plasma leakage has been linked to this. This lowers the venous return and delays the release of anaphylactic mediators, which cause redness, rash, and/or itching of the skin.

Cardiovascular hypersensitivity, rather than cardiac toxicity, seems to be the pathophysiologic basis of acute coronary syndromes, such as Kounis syndrome, myocarditis, and cardiac arrhythmias caused by cancer therapy. This is based on clinical and laboratory evidence. The chemotherapeutic agents that induce the hypersensitivity reactions are shown in Table 1.

Cardiovascular hypersensitivity should be used in conjunction with cardiac toxicity to describe the adverse events elicited by various chemotherapeutic agents, including monoclonal antibodies, and should not be overlooked.

**Figure 2:**
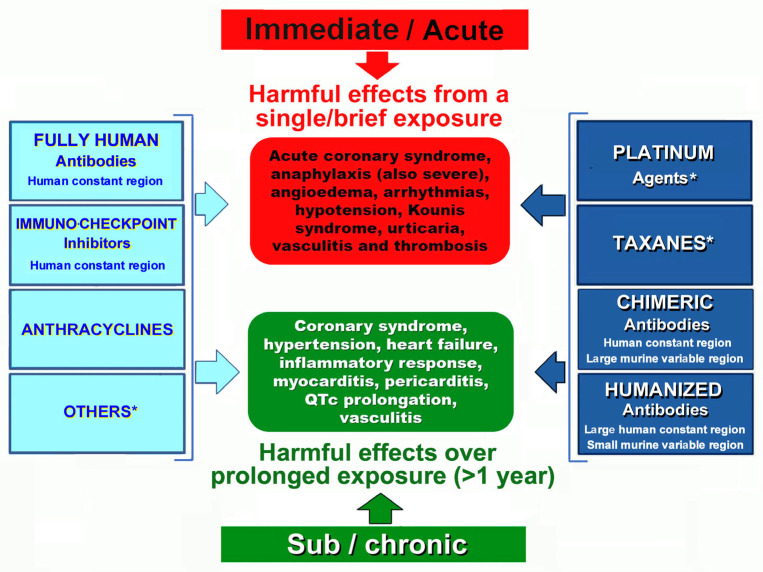
Hypersensitivity (immune-allergic) responses to cancer therapeutic agents. Some drugs can cause either immediate or delayed allergy.

The hypersensitivity reactions to chemotherapeutic agents are classified [11] as immediate, occurring in the first 6 h after the administration of treatment, or nonimmediate, usually occurring days or weeks later (Figure 2).

## 4. The Kounis Hypersensitivity-Associated Acute Coronary Syndrome

An initial diagnosis of serum pathology was made for allergic, hypersensitive, anaphylactic, or anaphylactoid reactions that are linked to cardiovascular symptoms. These reactions were classified as acute carditis, morphologic cardiac reactions, or rheumatic carditis with an unclear etiology. Kounis syndrome was later named after the initial thorough description of the allergic angina syndrome, which was first published in 1991 [12]. It was described as a coronary spasm that was a sign of endothelial dysfunction or microvascular angina, which resulted in an allergic acute myocardial infarction [13,14]. The inflammatory mediators released from mast cell degranulation and other interacting cells, such as T lymphocytes, macrophages, eosinophils, and platelets, during an allergic insult are the cause of this syndrome [15]. Chymase, which functions as a converting enzyme, along with tryptase, histamine, and arachidonic acid products, can all contribute to the acute ischemic event by causing coronary spasm, atheromatous plaque erosion or rupture, and platelet activation in the Kounis syndrome cascade. Along with the coronary arteries, Kounis syndrome can also affect the cerebral, mesenteric, and peripheral arteries.

Its incidence in patients who experience an allergic, hypersensitive, anaphylactic, or anaphylactoid insult ranges from 1.1% to 3.4% [16]. Initially believed to be an uncommon ailment, Kounis syndrome seems to be an underdiagnosed illness. Three types of this syndrome have been described so far [17]:

1. Type I or MINOCA type (myocardial infarction with nonobstructive coronary arteries), which affects 76.6% of patients with normal or nearly normal coronary arteries and is induced by histamine, chymase, or arachidonic acid products (leukotrienes, platelet-activating factor).
2. Type II, which affects 22.3% of patients with quiescent preexisting coronary disease and is induced by the same factors as type I plus platelet activation.
3. Type III, which affects 5.1% of patients and is induced by stent polymers, stent metals, eluted medications, dual antiplatelets, and environmental exposure in patients with stent thrombosis (subtype IIIa) and/or stent restenosis (subtype IIIb).

Kounis syndrome may be triggered by a variety of medications, including chemotherapeutic drugs, metals, foods, environmental exposures, and medical conditions (Table 2).

## 5. Hypersensitivity to Monoclonal Antibodies

There are three types of monoclonal antibodies used in the treatment of neoplastic, hematologic, or inflammatory diseases that include chimeric, humanized, fully human, and immune checkpoint inhibitors.

a. Chimeric monoclonal antibodies. The term “chimeric” derives from the Greek mythological monstrous, chimera. The chimera was one of the offspring of Typhon and Echidna and a sibling monster as Cerberus, and the Lernaean Hydra appeared as a fire-breathing goat-headed lion with a serpent-headed tail. These antibodies consist of human constant regions and murine variable regions. They are used as second-line drugs for the treatment of neoplastic, hematologic, and other chronic systemic inflammatory disorders, such as rheumatoid arthritis, ankylosing spondylitis, Crohn’s disease, and systemic vasculitis. They bind to the epidermal growth factor receptor and block receptor-dependent signal transduction pathways, such as anti-apoptosis, angiogenesis, and tumor metastasis. Both non-anti-TNF-α and anti-TNF-α chimeric monoclonal antibodies have been implicated in the development of immediate or delayed cardiac hypersensitivity reactions [40]. Chest pain, hypotension, severe anaphylaxis, and the acute hypersensitivity-associated coronary Kounis syndrome are the main adverse clinical manifestations [18,19,41,42]. Hypersensitivity reactions to rituximab may be due to either an immunoglobulin E (IgE) type I-mediated reaction or a massive cytokine release reaction [43].

b. Humanized monoclonal antibodies are produced by mouse–human hybrids and are made up mainly of a human sequence, with only a small portion of a mouse sequence in the complementarity-determining regions. In a recent report, the incidence and severity of anaphylaxis and hypersensitivity in trials of the intravenous humanized monoclonal antibodies, pertuzumab plus trastuzumab, or the fixed-dose combination of pertuzumab and trastuzumab for subcutaneous injection for HER2-positive breast cancer were analyzed. The authors recommended that, if such reactions occur, treatment should be delayed or abandoned and appropriate medical treatments should be administered [44].

c. Fully human monoclonal antibodies are derived from human sequences in both the constant and variable regions and are designed to minimize immunogenicity and maximize compatibility with the human immune system. Nivolumab is the only fully human monoclonal antibody that is used in practice and is well tolerated, but patients should also be warned of the possibility of serious hypersensitivity reactions, for which they should urgently see a physician for individualized evaluation [45].

d. Immune checkpoint inhibitors are drugs that inhibit proteins that stop the immune system from attacking the cancer cells. They act by targeting immunologic receptors on the surface of T-lymphocytes. These drugs can also cause hypersensitivity reactions. Such reactions are mild to moderate and include skin rash, itching, headache, fever, and nausea. While serious reactions are rare, they can be fatal without proper treatment. The incidence of such reactions with the use of avelumab was found to be 23.1% and desensitization protocols have been suggested to prevent these reactions [46]. The use of immune checkpoint inhibitors has been associated with myocarditis and acute myocardial infarction. Myocardial biopsies performed in six patients with immune checkpoint inhibitor-induced myocarditis revealed mast cells, hypereosinophilia, shrunken, and irregular cardiac myocytes, but were not associated with fibrosis [47]. The cytoplasm of mast cells is eosinophilic and contains varying amounts of lysosomes. Therefore, eosinophilic and histiocytic myocarditis are types of drug-induced hypersensitivity myocarditis [48], which were originally described by one of the authors of this paper. In a recent study of 3684 Asian Chinese patients receiving immune checkpoint inhibitors, 24 patients developed a myocardial infarction within the first 90 days [49]. In this study, sensitivity analyzes excluded patients with myocardial infarction-related death and the authors did not describe the patients’ clinical signs and symptoms. Therefore, the Kounis hypersensitivity myocardial infarction cannot be excluded. Moreover, cemiplimab is the only immune checkpoint inhibitor approved by the European Medicines Agency and about 4% of patients develop hypersensitivity reactions with its use. A severe hypersensitivity reaction to cemiplimab in a patient with cutaneous squamous cell carcinoma was successfully treated using a desensitization protocol [50].

## 6. Hypersensitivity to Cytotoxic Agents and Kounis Syndrome

Platinum-based chemotherapeutic agents, including cisplatin, carboplatin, oxaliplatin, nedaplatin (Japan), heptaplatin (Korea), and lobaplatin (China), impede DNA replication, thereby suppressing cancer cell division and proliferation. Every variety of platinum agent shares an identical platinum core, with a cross-reactivity of roughly 45%. All these agents have been implicated in the induction of hypersensitivity reactions, [6,51] usually of type I (Immediate type), but also rarely of type II (Cytotoxic type), type III (immune complex type), and type IV (delayed type). The range of hypersensitivity to cisplatin is 5–14%, to carboplatin it is 9–40%, and to oxaliplatin it is 10–25%.

Severe cardiac hypersensitivity reactions, such as the acute hypersensitivity-induced Kounis-type myocardial infarction, can occur in response to platinum agents and manifest as the usual symptoms of IgE/mast cell-mediated hypersensitivity reactions [20,21,22,23,24,25].

Taxanes, such as cabazitaxel, docetaxel, nab-paclitaxel, and paclitaxel, are complex alkaloid esters that provide broad-spectrum anticancer activity of solid malignancies. Their mechanism of action consists of the inhibition of cell division, chromatid separation, and growth, which ultimately leads to cell death. Hypersensitivity reactions are common and range from mild, severe, and fatal that is unresponsive to initial therapy. Approximately 30% of patients receiving taxanes have developed hypersensitivity reactions. Proposed mechanisms include IgE-mediated anaphylaxis with elevated tryptase levels, direct activation of mast cells and/or basophils, and complement activation [5]. Cross-reactivity in various types of taxanes increases to 50%. Nab-paclitaxel, which is similar to paclitaxel, contains human albumin particles instead of Cremophor El and the hypersensitivity reactions are fewer. Kounis syndrome associated with hypersensitivity has occurred in patients treated with paclitaxel administration [26,27]. Moreover, drug-coated balloons are used widely as a form of endovascular treatment for peripheral arterial disease. These balloons contain anti-proliferative drugs, such as paclitaxel, which improve vessel patency by reducing neointimal hyperplasia and restenosis. Indeed, acute coronary syndrome of Kounis type syndrome secondary to anaphylaxis after the inflation of a paclitaxel-coated balloon, which was used to treat recurrent superficial femoral artery stenosis, have been reported [28,29].

## 7. Other Anticancer Medicines Inducing Hypersensitive Reactions and Kounis Syndrome

**a. Alkylating agents** have the ability to attach an alkyl group to DNA. Alkylated DNA makes cancer cells much more sensitive to damaged DNA and, in this way, their proliferation is prevented. The alkylating agents, cyclophosphamide and ifosfamidem, are occasionally associated with hypersensitivity reactions [51].

**b. Antimetabolites**, which are also called nucleotide synthesis inhibitors, disrupt DNA synthesis by substituting for the natural metabolite or interfering with the production of a major nucleotide metabolite. The antimetabolite, capecitabine, is an orally available prodrug that is converted to 5-fluorouracil in tumor tissues and is used to treat metastatic colorectal cancer and breast cancer. Cardiac manifestations include angina, acute coronary syndrome, arrhythmias, myocarditis, and heart failure. It is not known whether these side effects are attributable to the accumulation of toxic metabolites or hypersensitivity. However, in a report of capecitabine-induced cardiac arrest due to ventricular fibrillation, the underlying pathology supported by immunological investigation showed Kounis type I hypersensitivity-related syndrome [30,31,32,33,34]. Moreover, 5-fluorouracil has been also reported to induce vasospasm of Kounis type syndrome [35,36,37].

**c. Anthracyclines** are chemotherapeutic agents that block DNA replication and the transcription of genes by binding DNA-forming adducts and crosslinks. Moreover, they can prevent the activity of DNA helicase and interfere with DNA strand separation. They can cause both cardiac toxicity and cardiovascular hypersensitivity. Up to 45% of patients with cancer can suffer hypersensitivity reactions during treatment if left without premedication [38,51]. However, with formulations such as pegylated liposomal doxorubicin or liposomal daunorubicin, the incidence of hypersensitivity reactions is around 9–14% [51,52]. The Kounis hypersensitivity-associated myocardial infarction has been reported as a side effect of epirubicin [53].

**d. Enzyme inhibitors** have been used as anticancer drugs because they inhibit the enzymes that involved in DNA replication and RNA transcription. The following four enzymes, PFK-2/FBPase-2, ATIC, LTA_4_H, and Jmjd6, are promising targets for the development of new anti-cancer drugs [54]. Irinotecan and topotecan are both enzyme inhibitors, which are used alone or combined with other agents to treat a variety of tumors. They belong to the DNA topoisomerase I inhibitor class of drugs [55,56]. Hypersensitivity reactions have been previously reported with the use of both drugs [57]. Topotecan was injected into sub-Tenon’s space in a fibrin sealant, which was used as an adjunct to laser therapy for small retinoblastoma tumors in 25 children (77 injections). Two children developed severe hypersensitivity reactions on their third sub-Tenon’s injection of topotecan in a fibrin sealant. The authors of this report suggested that such reactions could be the result of both the drug and fibrin glue [58].

**e. Protein kinase inhibitors** are a new approach to cancer treatment with targeted therapy. They can also cause both cardiac toxicity and cardiovascular hypersensitivity. These agents inhibit the protein kinase enzymes, which have the ability to phosphorylate proteins and modulate their function [59]. This dysfunction of protein kinase enzymes constitutes the basis of many cancers but also of several cardiovascular manifestations, such as ischemia/reperfusion injury, left ventricular remodeling, angiogenesis, and atherogenesis. Hypersensitivity reactions to protein kinase inhibitors can occur and desensitization strategies have been applied [60]. Urticaria and angioedema have been reported during their use. Some cases of Stevens-Johnson syndrome (flu-like symptoms, painful rash, and blisters) have been described with regorafenib, which is a vascular endothelial growth factor receptor inhibitor, palbociclib, which is a cyclin-dependent protein kinase inhibitor, and ribociclib [11]. Cross-reactivity between dabrafenib and vemurafenib has been reported, possibly due to their similar chemical structure [11].

**f. Tyrosine kinase inhibitors** are small molecules or peptides that have the ability to inhibit either cytosolic or receptor tyrosine kinases. They inhibit tyrosine kinases, which activate a variety of proteins via signal transduction cascades. These agents phosphorylate specific amino acids and modify cell growth, migration, differentiation, apoptosis, and death. This protein activation or inhibition causes dysregulation of the signal cascades, which can lead to malignancy and other pathologic conditions. Therefore, blocking these initial signals using tyrosine kinase inhibitors can prevent the aberrant action of the mutated or dysfunctional tyrosine kinases. In a study of 78 consecutive patients suffering from chronic myeloid leukemia who were treated with the third-generation tyrosine kinase inhibitor, three patients experienced myocardial infarction (3.8%) [61]. Imatinib was also reported to induce or promote cardiac dysfunction manifesting with cardiac arrythmias but, overall, cardiac side effects are rare [62]. The potential mechanisms of these side effects are unclear but hypersensitivity reactions cannot be excluded [63].

## 8. Transcatheter Arterial Chemoembolization for Patients with Cancer and Kounis Syndrome

A minimally invasive, nonsurgical technique called transcatheter embolization is used to reduce or eliminate the blood supply of a tumor [64]. Particle embolization and the injection of chemotherapy medications are combined in transarterial chemoembolization. In this manner, the size of the tumor is decreased, its symptoms are lessened, and its growth is postponed. The most often utilized single agents in transarterial chemoembolization are the chemotherapeutic drugs, doxorubicin, cisplatin, and epirubicin. As the most prevalent malignant tumor of the liver, hepatocellular carcinoma is specifically treated using this technique. However, these medications are combined with lipiodol, also known as iodized oil, which is a vehicle for the delivery of chemotherapeutic medications that remain in the tumor nodules following injection into particular hepatic artery branches. This procedure is often complicated by a syndrome called postembolization syndrome. The symptoms of this syndrome include fever, vomiting, nausea, and abdominal pain and may affect 60% to 70% of individuals undergoing transarterial chemoembolization. Hepatic insufficiency and, in rare cases, lipiodol embolism to the brain and lung are also serious side effects. Transcatheter arterial chemoembolization is most useful as a liver transplant bridge and as the initial line of treatment for inoperable hepatocellular carcinoma. Additionally, Kounis syndrome was reported in a 79-year-old man with hepatocellular cancer 30 min following successful transcatheter arterial chemoembolization [65]. After receiving an injection of ethylester of iodinated poppy-seed oil fatty acid, iopamidol, and an iodinated contrast medium, the patient started to complain of anterior chest pain and eventually lost consciousness. An inferior myocardial infarction was diagnosed by a twelve-lead ECG that displayed a full atrioventricular block with junctional escape rhythm and considerable ST segment elevation in the II, III, and aVF leads. On the other hand, the right coronary artery angiography showed no signs of blockage or stenosis. This patient may have experienced a coronary artery spasm after receiving two allergenic substances, one of which may have been a chemotherapeutic medication (the authors did not specify which one) combined with iodinated contrast media. Indeed, the theory that patients who are simultaneously exposed to more than one allergen may exhibit more symptoms than those who are only sensitive to one allergen is supported by clinical research [39]. When a patient is concurrently exposed to the appropriate antigens, IgE antibodies with varying specificities can have cumulative effects and in small, even subthreshold, quantities, this can cause the release of cell mediators [66].

## 9. Conclusions

Since the treatment of cancer and cardiovascular illness have become tightly associated, it is anticipated that the incidence of major cardiovascular problems associated with cancer treatment will rise over time. It is crucial to have dedicated cardiovascular clinics to treat patients with cancer with heart failure or cardiac hypersensitivity. These clinics should offer professional pre-treatment evaluation, monitoring, and treatment to ensure that cancer treatment proceeds as planned.

The relevant medical faculties of cardiology, allergy, and oncology have issued excellent guidelines for the treatment and prevention of cancer therapy-related cardiovascular toxicity criteria and the hypersensitivity reactions of chemotherapeutic agents. However, the Spanish multidisciplinary research network for allergic diseases has issued guidelines on the hypersensitivity reactions to cancer chemotherapy, emphasizing the need for diagnosis, management, and desensitization procedures without referring to cardiotoxicity [67]. Likewise, the European Network on Drug Allergy and Drug Allergy Interest Group of the European Academy of Allergy and Clinical Immunology have organized a task force to provide data and recommendations regarding the allergological work on the field of hypersensitivity reactions to chemotherapy without referring to cardiotoxicity [68,69,70]. In the 2022 European Society of Cardiology (ESC) Guidelines on cardio-oncology, in collaboration with the European Hematology Association (EHA), the European Society for Therapeutic Radiology and Oncology (ESTRO), and the International Cardio-Oncology Society (IC-OS), the hypersensitivity reactions and the Kounis hypersensitivity-associated coronary syndrome are absent, despite many reports in the medical literature emphasizing cardiovascular hypersensitivity and Kounis syndrome as severe side effects of cancer therapy [8]. We believe that closed coordination and understanding among all these excellent organizations are necessary for both researchers and practicing physicians.

For the benefit of patients and society, cardiac toxicity, cardiovascular hypersensitivity, and Kounis syndrome are crucial triplets in cardio-oncology that every general practitioner and specialist should be aware of.

## Figures and Tables

**Table 1:** Chemotherapeutic agents inducing hypersensitivity reactions.

Monoclonal antibodies
Chimeric: Infliximab, Rituximab, Cetuximab, Brentuximab, Abciximab, Basiliximab
Humanized: Trastuzumab, Pertuzumab, Benacizumab
Human: Nivolumab
Immune checkpoint inhibitors: Avelumab, Atezolizumab, Cemiplimab, Durvalumab, Ipilimumab, Nivolumab, Pembrolizumab
**Platinum agents**
Carboplatin, Cisplatin, Oxaliplatin, Nedaplatin (Japan), Heptaplatin (Korea), Lobaplatin (China)
**Taxanes**
Cabazitaxel, Docetaxel, Nab-Paclitaxel, Paclitaxel
**Other**
Alkylating agents: Cyclophosphamide, Ifosfamidem, Melphalan, Busulfan, Dacarbazine, Thiotepa, Carmustine
Antimetabolites: Fluorouracil, Cabecitabine, Floxuridine, Cytarabine, Deoxycytidine analogues: Gemcitabine
Anthracyclines: Daunorubicin, Doxorubicin, Epirubicin, Idarubicin, Mitoxantrone, Valrubicin.
Enzyme inhibitors: Irinotecan, Topotecan
Protein kinase inhibitors: Afatinib, Alectinib, Axitinib, Bosutinib, Cabozantinib, Ceritinib, Cobimetinib, Crizotinib…
Tyrosine kinase inhibitors: Imatinib, Cetuximab, Erlotinib, Gefitinib

**Table 2:** Hypersensitivities to chemotherapeutic agents and Kounis syndrome. All patients received basic treatment with corticosteroids and antihistamines, and symptomatic treatment for accompanying diseases.

Country	First Author	Drug	Type of Cancer	Administration	Reference
Germany	Gori T	Rituximab	Hairy cell leukemia	Infusion	[18]
Puerto Riko	Diaz-RodriguezPE	Infliximab	Crohn’s disease	Infusion	[19]
Italy	Boroni M	Carboplatin	Lung adenocarcinoma	Infusion	[20]
USA	Tambe V	Carboplatin	Esophangeal adenocarcinoma	Infusion	[21]
Italy	Oneglia C	Cisplatin	Ovarian cancer	Infusion	[22]
Taiwan	Chang PH	Oxaliplatin	Sigmoid adenocarcinoma	Infusion	[23]
Italy	Albanes M	Oxaliplatin	Colorectal carcinoma	Infusion	[24]
Greece	Kounis N	Cisplatin	Nasopharynx carcinoma	Infusion	[25]
Australia	Wang B	Paclitaxel	Lung adenocarcinoma	Infusion	[26]
Australia	Wang B	Paclitaxel	Lung adenocarcinoma	Infusion	[27]
Australia	Narroway HG	Paclitaxel	Femoral artery stenosis	Ballon inflation	[28]
England	Lake E	Paclitaxel	Femoral artery stenosis	Ballon inflation	[29]
USA	Kido K	Capecitabine	Colon adenocarcinoma	Oral	[30]
Greece	Kounis N	Capecitabine	Colorectal cancer	Infusion	[31]
England	Scott PA	Capecitabine	Rectal cancer	Oral	[32]
England	Coughlin S	Capecitabine	Stomach cancer	Infusion	[33]
Greece	Tsiamis E	Capecitabine	Colorectal cancer	Infusion	[34]
Italy	Canale ML	5-fluorouracil	Colorectal cancer	Infusion	[35]
USA	Tajik R	5-fluorouracil	Colon cancer	Infusion	[36]
Turkey	Karabay CY	5-fluorouracil	Signet-ring cell carcinoma	Slow infusion	[37]
China	Liang HZ	Epirubicin	Bladder cancer	Slow injection	[38]
Japan	Lyonaga T	Iopamidol	Hepatocellular carcinoma	Embolization	[39]

## Data Availability

Data are contained within the article.
